# Access to specialist palliative care and health care service utilization during the final year of life among patients with kidney disease

**DOI:** 10.1093/ckj/sfag111

**Published:** 2026-04-09

**Authors:** Hanna-Riikka Lehto, Maarit Wuorela, Satu Ahtiluoto, Mikko Nuutinen, Tiina Saarto, Timo Carpén, Outi Akrén

**Affiliations:** Department of General Medicine, Division of Palliative Medicine, University of Turku, Turku, Finland; Department of Medicine/Division of Nephrology, Turku University Hospital and the Wellbeing Services County of Southwest, Turku, Finland; Palliative Care Center, Comprehensive Cancer Center, Helsinki University Hospital and University of Helsinki, Helsinki, Finland; Nordic Healthcare Group, Helsinki, Finland; Palliative Care Center, Comprehensive Cancer Center, Helsinki University Hospital and University of Helsinki, Helsinki, Finland; Palliative Care Center, Comprehensive Cancer Center, Helsinki University Hospital and University of Helsinki, Helsinki, Finland; Department of General Medicine, Division of Palliative Medicine, University of Turku, Turku, Finland; Palliative Care Center, Comprehensive Cancer Center, Helsinki University Hospital and University of Helsinki, Helsinki, Finland

**Keywords:** cancer, end-of-life care, health care utilization, malignant kidney disease, non-malignant kidney disease, palliative care

## Abstract

**Background:**

Despite the heavy symptom burden and progressive nature of non-malignant kidney diseases, access to palliative care services may be limited. We evaluated access to specialist palliative care (SPC), and its effect on health care utilization among patients with non-malignant and malignant kidney disease during their final year of life.

**Methods:**

This retrospective cohort study examined causes of death among ≥18-year-old individuals in Finland in 2019 using the National Causes of Death Register. Data on access to SPC, emergency department contacts, and hospitalizations were collected from the National Care Register for the final year of life.

**Results:**

Five hundred eighty-five patients had non-malignant kidney disease (54.8% females, mean age 84 years at the time of death) and 706 patients had malignant kidney disease (35.3%, 77.2 years, respectively). Of the patients with non-malignant kidney disease, only 54 (9.1%) had access to SPC services compared to 195 (27.6%) with malignant kidney disease (*P* < .001). Within patients with malignant kidney disease, those who had access to SPC died at home more often (10.6% vs. 5.9%; *P* = .049), had fewer emergency department contacts (19.7% vs. 32.9%), and had a lower proportion of hospitalizations (16.4% vs. 37.2%; *P* < .001) and readmissions to secondary care (4.6% vs. 10.6%; *P* = .025) when compared to those without SPC access. No difference with regard to SPC access was seen among patients with non-malignant kidney disease.

**Conclusion:**

While SPC demonstrated benefits in malignant kidney disease patients’ health care utilization, access was markedly limited for patients with non-malignant conditions, underscoring the need for improvements in service provision.

## Graphical Abstract

**Graphical Abstract:**
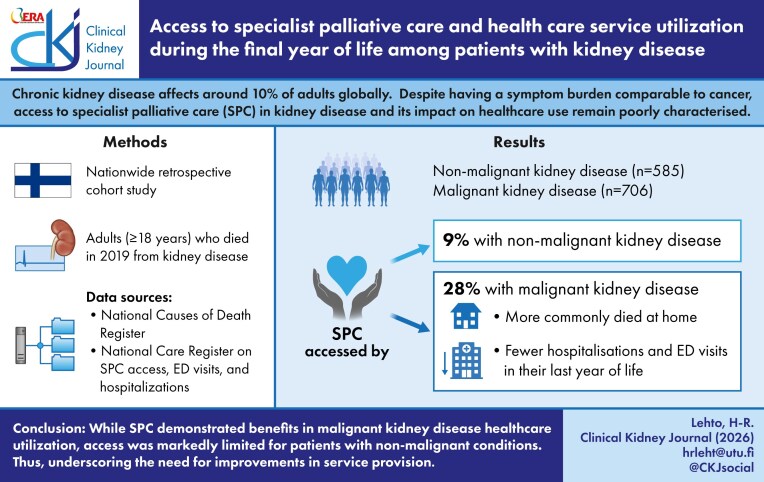

KEY LEARNING POINTS **What was known:**

- Chronic kidney diseases affect approximately 10% of the adult population globally and are projected to become the fifth leading cause of death by 2040, yet their symptom burden and care needs remain under-recognized compared to malignant conditions.
- Patients with end-stage non-malignant kidney disease experience symptom loads comparable to those of cancer, but access to specialist palliative care (SPC) is poorly documented and inconsistently integrated into nephrology care.
- Despite recommendations to offer palliative care for non-malignant kidney diseases, population-level data on SPC access and its impact on healthcare utilization in the final year of life are lacking.

**This study adds:**

- Patients with non-malignant kidney disease had significantly lower (<10%) access to specialist palliative care (SPC) than those with malignant kidney disease (28%).
- Emergency department visits and hospitalizations were frequent at the end of life, especially among patients with non-malignant kidney disease, indicating unmet care needs.
- This study reveals national disparities in SPC access and highlights the urgency of integrating palliative care into nephrology for patients with non-malignant kidney disease.

**Potential impact:**

- This study underscores the importance of educating clinicians who treat non-malignant kidney disease about patients’ limited prognosis and palliative care needs, to improve care quality and reduce avoidable end-of-life hospitalizations.
- Increasing awareness among clinicians may help overcome misconceptions and barriers that limit timely access to palliative care in non-malignant kidney disease.
- Policy efforts should prioritize expanding hospital-at-home services and integrating nephrology and palliative care teams, as well as promoting equitable end-of-life care across patient populations to reduce hospital costs and align care with patient preferences.

## INTRODUCTION

The burden of non-malignant renal diseases, such as chronic kidney disease, has increased significantly in recent decades [1]. Currently, chronic kidney diseases rank as one of the 10 leading causes of death worldwide [2, 3]. The aging population and increasing obesity rates are expected to increase the prevalence of chronic kidney disease and elevate it into the fifth leading cause of death by 2040 [4]. This increasing prevalence poses significant challenges for health care services.

Non-malignant chronic kidney diseases often progress silently until nephron depletion reaches advanced stages, leading patients to suffer from fatigue, fluid retention, electrolyte imbalances, pain, and decreased urine output [5]. The symptom burden among patients with end-stage kidney disease is equivalent to that for patients with end-stage cancer [6, 7]. Patients with end-stage kidney disease often suffer from multiple comorbidities and increasing frailty, leading to low quality of life (QOL) and frequent hospitalizations [8].

Palliative care is a medical specialty dedicated to alleviating symptoms and improving QOL, irrespective of the patient’s remaining lifespan [9]. Traditionally, cancer patients have dominated palliative care service utilization. However, adults with non-malignant diseases are estimated to constitute around 65% of those needing palliative care [10]. For patients with non-malignant kidney disease, the combination of high symptom burden and limited lifespan after dialysis initiation—approximately 30 years shorter at age 40 and 17–20 years shorter at age 60 compared with the general population [11]—underscores the complexity of care and the need for comprehensive support. These challenges have prompted the development of programs that integrate palliative care with nephrology [12–14].

Although the need for palliative care is growing and the National Kidney Foundation [15] recommends offering it to all chronic kidney disease patients regardless of stage, population-level access remains poorly understood. Also, the impact of specialist palliative care (SPC) contact on healthcare utilization in the final year of life is unknown.

Therefore, we aimed to evaluate access to SPC nationwide and use of emergency departments and hospitalizations during the final year of life among patients with non-malignant kidney disease. Secondly, we aimed to compare health care service utilization between patients with non-malignant and malignant kidney disease to inform future directions for developing palliative services for patients with non-malignant kidney disease at the population level.

## MATERIALS AND METHODS

### Design and study cohort

This retrospective decedent cohort study used national health care registers to examine SPC access and its impact on service utilization during the final year of life among adults (≥18 years) who died in 2019 from non-malignant renal disease or malignant kidney or urothelial cancer.

We collected deaths among adult patients in Finland during 2019 from the National Causes of Death Register [16]. The process of compiling this data has previously been described in detail [17]. In Finland, the clinician responsible for care at the time of death identifies the primary cause of death using the International Classification for Diseases version 10 (ICD-10). In addition, up to four contributing causes of death can be listed using ICD-10 codes. These are recorded in the death certificate along with the service unit, residency, and social security details. The certificates are later approved by Statistics Finland.

We included 1303 patients with ICD-10 codes indicating non-malignant kidney disease as the primary cause of death (Supplementary Table S1). In Finland, the primary cause of death is defined as the underlying chronic condition, while infections or acute complications can be recorded as the immediate cause; therefore, only primary causes were examined. Malignant kidney diseases included ICD-10 codes for bladder, urothelial, and kidney cancers. For non-malignant kidney diseases, ICD-10 codes for kidney failure caused by hypertension, diabetes, and other causes were included. The death certificate data on the date of death, latest municipality of residence, and place of death were collected. Two patients who died outside Finland, with no health care usage during the final year of life, were excluded from our analyses.

### Health care service structure

Finland, a country of 5.6 million people, has a universal healthcare system covered mostly by taxes. In 2019, over 300 municipalities organized primary care [18]. Secondary care, both outpatient and in-hospital, was provided by five academic university hospitals, along with 15 central hospitals [18]. The medical care provided in these academic hospitals and central hospitals, at tertiary and secondary levels, respectively, is here referred to as secondary care. All university hospitals had a palliative care service and 12 of the 15 central hospitals had palliative outpatient units in 2019 [18]. The unique feature of the Finnish healthcare system is general practitioner or geriatric-led in-patient ward units located in primary care [18, 19]. These units cover most of the basic palliative care, along with hospital-at-home services (hospital-level care at home with physician involvement) and home services (mostly nurse-led services). Some of these wards and hospital-at-home services are further specialized to provide SPC. Institutional long-term care is provided mostly for the elderly and patients with disabilities in nursing homes [18]. All healthcare organizations are mandated by Finnish law to report all care provided to patients in the nationwide Care Register for Health Care.

### Specialist palliative care

SPC is provided both in the primary and secondary care hospitals in Finland. In this study, SPC services offered either in primary or secondary care services are combined and referred to as SPC. These services can be provided as any combination of outpatient contacts, palliative care team consultations in secondary care, hospital-at-home services, and inpatient or hospice care.

### The health care service utilization and data linkage

We used the Care Register for Health Care, maintained by the Finnish Institute for Health and Welfare, to collect all health care contacts that occurred in emergency departments, hospitalizations in primary care and specialist level, outpatient contacts, and use of dialysis (ICD-10 codes Z49.1 and Z49.2). Each of the health care service sites in Finland has a unique identification code. Data on different administrative registers were combined using the social security code unique to each resident.

### Outcomes

Place of death, acquired from the death certificate, was categorized as home, long-term care, or hospital (secondary and primary care ward combined). Each SPC unit as a place of death was identified using its unique identification code. Health care utilization was evaluated with the proportion of patients receiving care, average length of stay, and readmissions at each time interval prior to death.

### Ethical statement

This study was part of a Project on Quality Information on Palliative Care and End-of-life Care and was conducted in collaboration with the Finnish Institute for Health and Welfare. Approval for the study was obtained from the Finnish Institute for Health and Welfare (THL/908/6.02.00/2021). According to the Finnish legislation, this study was considered exempt from separate ethical permission as data from deceased patients were used. The numbers of less than three persons are reported as <3 due to Finnish and EU privacy legislation. We followed the STROBE checklist to report this study [20].

### Statistical methods

Descriptive statistics were generated with frequencies and percentages for categorical variables, and with means, medians, standard deviations, and interquartile range, as appropriate, for continuous variables. Differences in health care utilization and other variables between groups were examined using Pearson’s chi-squared test with Fisher’s exact test for categorical variables, and Mann-Whitney U-test for continuous variables. Patient characteristics were evaluated using means with standard deviations and frequencies. Statistical significance was set at *P* < .05. All statistical analyses were performed using SPSS version 29 (IBM Corporation, SPSS Inc., NY, USA).

## RESULTS

A total of 585 patients with non-malignant kidney disease (54.8% females) and 706 patients with malignant kidney disease (35.3% females) died in 2019. Patients with non-malignant kidney disease were on average 7 years older at the time of death compared to patients with malignant kidney disease (84.1 years vs. 77.1 years; *P* < .001, Table 1). A higher proportion of patients with non-malignant kidney disease lived in rural areas compared to patients with malignant kidney disease, who more commonly lived in urban areas (Table 1). The most common contributing causes of death among patients with non-malignant kidney disease were dementia (16%), atrial fibrillation and flutter (14.8%), heart failure and valvular diseases (12.3%), diabetes (13.4%), and infectious diseases (11.4%). The corresponding proportions for patients with malignant kidney disease are shown in Table 1.

**Table 1:** Patient characteristics at the time of death and divided between cancers or non-malignant based on the primary cause of death.

	Patients with non-malignant kidney disease	Patients with malignant kidney disease	*P*-value
Number of patients, *n*	595 (45.7%)	706 (54.3%)	
Females, *n* (%)	326 (54.8%)	249 (35.3%)	<.001
Age at death (mean ± SD)	84.1 ± 9.7	77.2 ± 10.7	<.001
**Municipality type at time of death**			
Urban	352 (59.2%)	459 (65.0%)	.034
Semi-urban	110 (18.5%)	132 (18.7%)	.943
Rural	133 (22.4%)	115 (16.3%)	.006
**Prevalence of contributory causes of death** ^**^b^**^			
None	145 (24.4%)	429 (60.8%)	<.001
Dementia	95 (16.0%)	51 (7.2%)	<.001
Infectious diseases	67 (11.4%)	22 (3.1%)	<.001
Diabetes	80 (13.4%)	35 (5.0%)	<.001
Hypertension	55 (9.2%)	43 (6.1%)	.035
Coronary artery disease	29 (4.9%)	63 (8.9%)	.005
Heart failure and valvular diseases	73 (12.3%)	26 (3.7%)	<.001
Atrial fibrillation or flutter	88 (14.8%)	26 (3.7%)	<.001
Stroke or intracerebral hemorrhage	38 (6.4%)	21 (3.0%)	.005
Peripheral artery disease	23 (3.9%)	8 (1.1%)	.002
Chronic obstructive pulmonary disease	25 (4.2%)	26 (3.7%)	.669
Cancer	38 (6.4%)	43 (6.1%)	.908
Chronic kidney disease	9 (1.5%)	17 (2.4%)	.321
Palliative care identification (defined as prevalence of ICD-10 code Z51.5) by the time of death	57 (9.6%)	385 (54.5%)	<.001
Average length of stay in hospital during the final 2 weeks of life ± SD	9.6 ± 4.7	10.5 ± 4.4	.001
**Place of death**			
Hospital^b^	458 (77.0%)	598 (84.7%)	<.001
At the palliative care ward at the time of death (included in hospital^b^)	8 (1.3%)	42 (5.9%)	<.001
Home	33 (5.5%)	50 (7.1%)	.306
Died under specialist palliative hospital-at-home care	0 (0%)	8 (1.1%)	.009
Long term care facility	104 (17.5%)	58 (8.2%)	<.001

a Municipality types are classified based on number of population living in the urban settlements and size of the largest city in the area (Statistical grouping of municipalities 2024 | Statistics Finland)

b ICD-10 codes used for contributory causes for death are shown in the Supplementary Table S2

### Location of death

Most deaths occurred in hospital: 77% of the patients with non-malignant kidney disease died in hospital compared to 84.5% of the patients with malignant kidney disease (*P* < .001; Table 1). A higher proportion of the patients with non-malignant kidney disease died in long-term facilities compared to patients with malignant kidney disease (17.5% vs. 8.2%; *P* < .001), while no difference was seen in the prevalence of death at home (5.5% vs. 7.1%; *P* = ns).

### Health care utilization during final year of life

**Figure 1:**
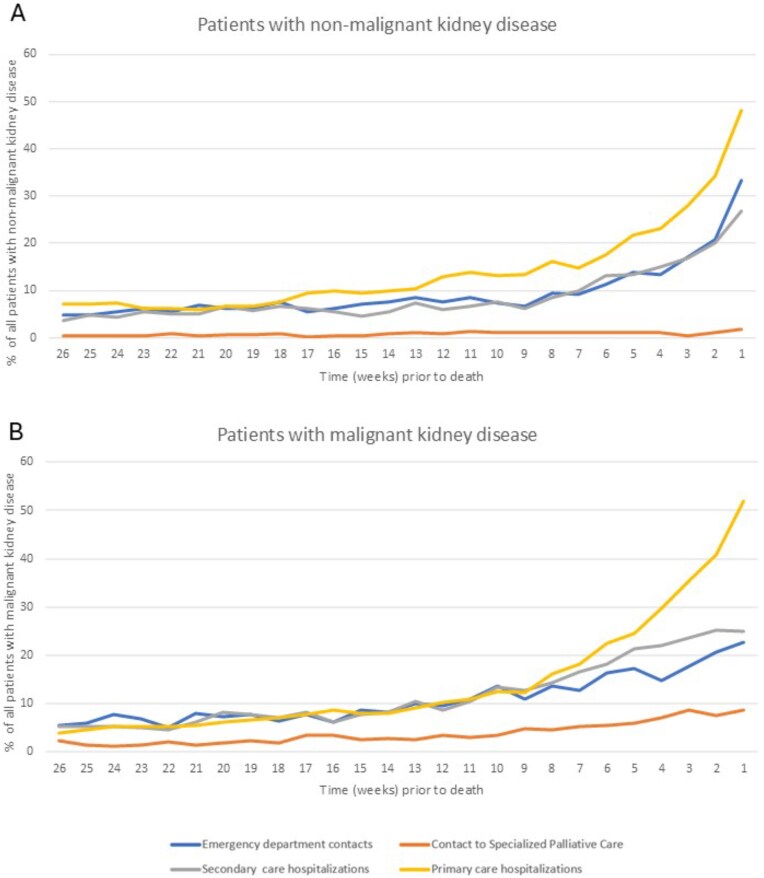
Non-cumulative proportion of patients with hospitalization and contacts to emergency department and specialist palliative care among patients with non-malignant and malignant kidney disease.

**Figure 2:**
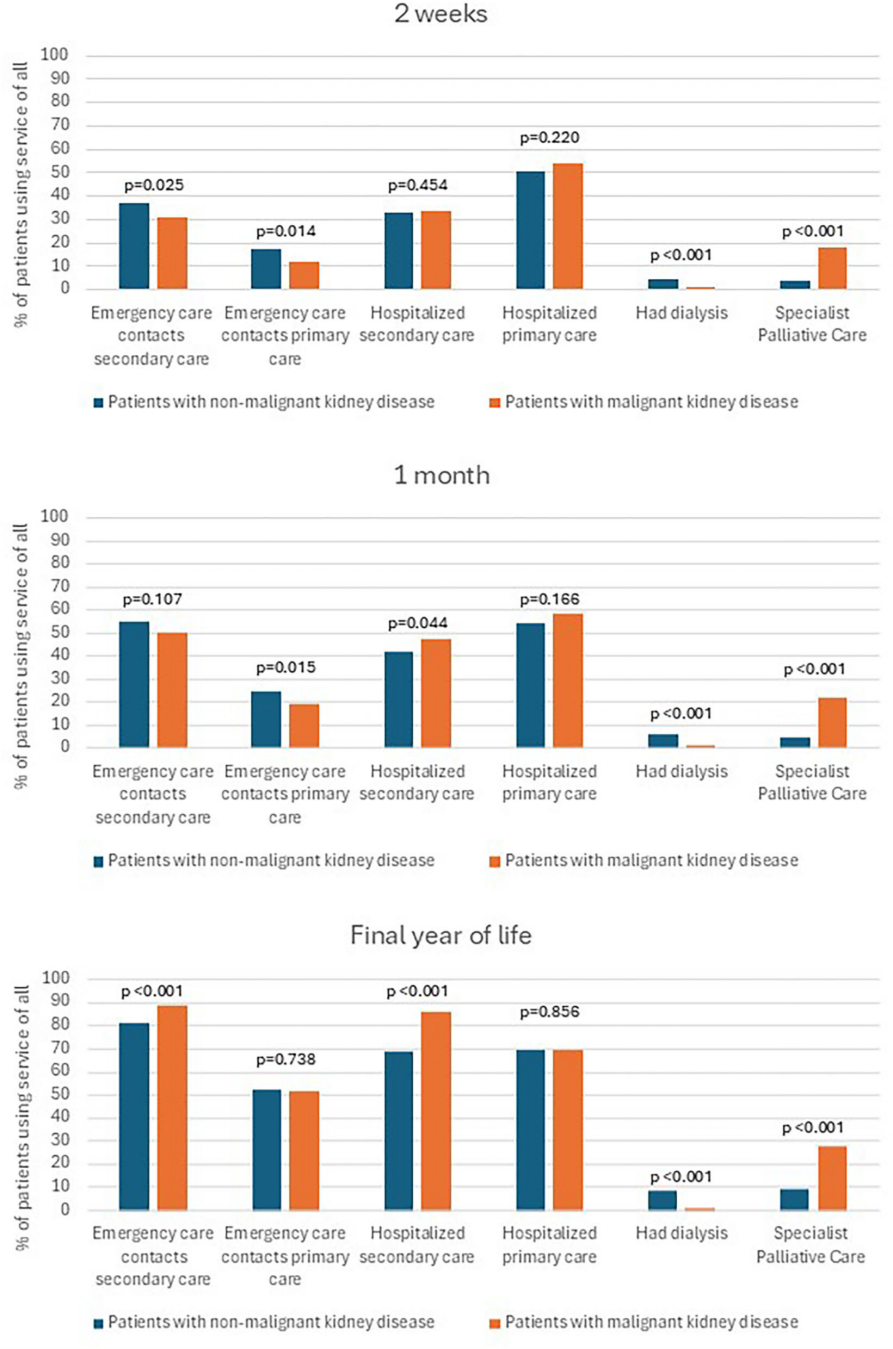
Cumulative proportion of patients using different types of health care services during the last 2 weeks, last 1 month, and final year of life.

Utilization of healthcare services increased toward the end of life (EOL) among both patient groups. Figure 1 shows non-cumulative proportions of patients in each group and their service utilization each week before death during the final 6 months of life. The proportion of patients admitted to secondary care hospital decreased toward EOL among those with malignant kidney disease, whereas the proportion increased among patients with non-malignant disease (Fig. 1). The proportions of patients using different health care services at the time points of 2 weeks, 1 month, and 1 year before death are shown in Fig. 2. During the final year of life, malignant patients had more secondary care hospitalizations and readmissions to healthcare, while primary care usage was similar (Table 2).

**Table 2:** Cumulative numbers of total health care contacts, length of stay, and readmissions among patients with non-malignant and malignant kidney disease at the time points of 2 weeks, 1 month, and during the final year of life.

	Patients with non-malignant kidney disease	Patients with malignant kidney disease	*P*-value
	*n* = 595	*n* = 706	
**2 weeks**			
**Emergency department (ED) contacts**			
Total number of ED contacts	452	461	
Median number (range) of ED contacts	1 (1–9)	1 (1–18)	.916
Patients with readmission to ED, *n* (%)	99 (16.6%)	93 (13.2%)	.085
Median number (range) of ED readmissions	1 (1–8)	1 (1–17)	.140
**Hospitalizations**			
***Secondary care***			
Total number of inpatient days	1191	1593	
Patients with hospitalization, *n* (%)	197 (33.1%)	237 (33.6%)	.454
Length of stay (median, IQR)	4 (5)	4 (5)	.585
Patients with readmission, *n* (%)	38 (6.4%)	68 (9.6%)	.041
Median number (range) of readmissions	1 (1–3)	1 (1–3)	.660
***Primary care***			
Total number of inpatient days	2668	3750	
Patients with hospitalization, *n* (%)	322 (54.1%)	413 (58.5%)	.116
Length of stay (median, IQR)	9.0 (11.25)	10.0 (18.50)	.045
Patients with readmission, *n* (%)	89 (15.0%)	116 (16.4%)	.492
Median number (range) of readmissions	1 (1, 1)	1 (1, 2)	.429
**1 month**			
**Emergency department contacts**			
Total number of ED contacts	738	839	
Median number (range) of ED contacts	1 (1–10)	1 (1–24)	.694
Patients with readmission to ED, *n* (%)	178 (29.9%)	185 (26.2%)	.154
Median number (range) of ED readmissions	1 (1–9)	1 (1–23)	.299
**Hospitalizations**			
***Secondary care***			
Total number of inpatient days	2139	3182	
Patients with hospitalization, *n* (%)	250 (42.0%)	337 (47.7%)	.044
Length of stay (median, IQR)	4.83 (5.83)	4.50 (5.5)	.692
Patients with readmission, *n* (%)	89 (15.0%)	135 (19.1%)	.055
Median number (range) of readmissions	1 (1–4)	1 (1–5)	.201
***Primary care***			
Total number of inpatient days	4509	6539	
Patients with hospitalization, *n* (%)	322 (54.1%)	413 (58.5%)	.116
Length of stay (median, IQR)	9.0 (11.25)	10.0 (18.50)	.045
Patients with readmission, *n* (%)	89 (15.0%)	116 (16.4%)	.492
Median number (range) of readmissions	1 (1–3)	1 (1–4)	.429
**1 year**			
**Emergency department contacts**			
			
Total number of ED contacts	2918	3864	
Median number (range) of ED contacts	4 (1–62)	4 (1–7)	.297
Patients with readmission to ED, *n* (%)	468 (77.0%)	591 (83.7%)	.002
Median number (range) of ED readmissions	4 (1–61)	4 (1–70)	.980
**Hospitalizations**			
***Secondary care***			
Total number of inpatient days	7792	10 918	
Number (%) of patients hospitalized	411 (69.1%)	608 (86.1%)	<.001
Length of stay (median, IQR)	4.0 (5.13)	4.0 (3.50)	.120
Number (%) of patients with readmission	296 (49.7%)	493 (69.8%)	<.001
Median number (range) of readmissions	2.0 (1–13)	3.0 (1–16)	.019
***Primary care***			
Total number of inpatient days	15 701	16 602	
Patients with hospitalization, *n* (%)	416 (69.9%)	490 (69.4%)	.856
Length of stay (median, IQR)	9.79 (11.17)	10.0 (12.81)	.784
Patients with readmission, *n* (%)	295 (49.6%)	309 (43.8%)	.039
Median number (range) of readmissions	2 (1–12)	2 (1–13)	.038

### Health care service utilization during the final 2 weeks of life

A higher proportion of patients with non-malignant kidney disease had emergency department contacts to secondary care (37.1%) compared to those with malignant disease (31.2%; *P* = .025) during the last 2 weeks before death. Similarly, emergency department contacts at primary care were more frequent among patients with non-malignant disease (17.0%) than among patients with malignant disease (12.0%; *P* = .014) (Fig. 2). During the final 2 weeks of life, 68.9% (*n* = 410) of patients with non-malignant kidney disease and 77.9% (*n* = 550) of patients with malignant kidney disease were hospitalized in either secondary or primary care wards, and the combined average length of stay in hospital was 9.6 days (±SD 4.7) among patients with non-malignant kidney disease and 10.5 days (±SD 4.4 days) (*P* = .001) among patients with malignant kidney disease.

### Use of specialist palliative care during final year of life

Only 9.1% (*n* = 54) of the patients with non-malignant kidney disease had access to SPC services during the final year of life, compared to 27.6% (*n* = 195) of the malignant kidney disease patients (*P* < .001). Median time to first SPC contact was 100.5 (IQR 200) days before death among patients with non-malignant disease, compared to 56 (IQR 130) days among those with malignant kidney disease (*P* = .049).

When further exploring the effects of SPC services on the use of other health services at the time points of 2 weeks and 1 month, we excluded those patients who received their first SPC service contact at any time after the studied time point. Among patients with malignant disease, access to SPC decreased the proportion of patients having contact to emergency departments in secondary care, while increasing emergency department use in primary care. At 2 weeks and 1 month, among patients with malignant kidney disease, SPC services also decreased the proportion of patients hospitalized at secondary care and the proportion of patients readmitted to secondary care (Table 3). At 1 month prior to death, among patients with non-malignant kidney disease, a higher proportion of patients with SPC used primary health care emergency departments. No effect was seen in secondary care utilization among those with access to SPC (Table 3).

**Table 3:** Comparison of health care service utilization between patients who had specialist palliative care contact prior to studied timepoint and those without specialist palliative care contact, among patients with non-malignant and malignant kidney disease.

	Patients with non-malignant kidney disease			Patients with malignant kidney disease		
	Specialist palliative care contact	No specialist palliative care contact	*P*-value	Specialist palliative care contact	No specialist palliative care contact	*p*-value
**2 weeks**						
Total number of patients^a^	45	541		152	511	
**Emergency department (ED) contacts**						
***Secondary care***						
Patients with ED contacts, *n* (%)	14 (31.1%)	205 (37.9%)	.425	30 (19.7%)	168 (32.9%)	.002
Patients with readmissions to ED, *n* (%)	4 (8.9%)	42 (7.8%)	.772	4 (2.6%)	36 (7.0%)	.051
***Primary Care***						
Patients with ED contacts, *n* (%)	12 (26.7%)	87 (16.1%)	.094	23 (15.1%)	52 (10.2%)	.108
Patients with readmissions to ED, *n* (%)	8 (17.8%)	28 (5.2%)	.004	10 (6.6%)	24 (4.7%)	.401
**Hospitalizations**						
***Secondary care***						
Patients with hospitalizations, *n* (%)	14 (31.1%)	181 (33.3%)	.875	25 (16.4%)	190 (37.2%)	<.001
Length of stay, median (IQR)	10.5 (8.25)	3.5 (5.0)	<.001	3.5 (7.25)	4.0 (4.63)	.203
Patients with readmissions, *n* (%)	2 (4.4%)	36 (6.7%)	.759	7 (4.6%)	54 (10.6%)	.025
***Primary care***						
Patients with hospitalizations, *n* (%)	19 (42.2%)	277 (51.2%)	.279	74 (48.7%)	2 (57.1%)	.077
Length of stay, median (IQR)	14.0 (12.0)	8.0 (10.0)	.662	12.5 (9.0)	10.5 (8.5)	.361
Patients with readmissions, *n* (%)	2 (4.4%)	35 (6.5%)	.759	4 (2.6%)	38 (7.4%)	.036
**1 month**						
Total number of patients^b^	40	541		131	511	
**Emergency department (ED) contacts**						
***Secondary care***						
Patients with ED contacts, *n* (%)	17 (42.5%)	302 (55.8%)	.138	55 (42.0%)	259 (50.7%)	.079
Patients with readmissions to ED, *n* (%)	5 (12.5%)	75 (13.9%)	1.000	10 (7.6%)	90 (17.6%)	.004
***Primary Care***						
Patients with ED contacts, *n* (%)	16 (40.0%)	127 (23.5%)	.034	33 (25.2%)	83 (16.2%)	.022
Patients with readmissions to ED, *n* (%)	11 (27.5%)	55 (10.2%)	.003	16 (12.2%)	38 (7.4%)	.110
**Hospitalizations**						
***Secondary care***						
Patients with hospitalizations, *n* (%)	15 (37.5%)	229 (42.3%)	.620	38 (29.0%)	261 (51.1%)	<.001
Length of stay, median (IQR)	11.0 (12.5)	4.33 (5.58)	.032	3.0 (6.38)	5.0 (6.0)	.287
Patients with readmissions, *n* (%)	6 (15.0%)	83 (15.3%)	1.000	12 (9.2%)	106 (20.7%)	.002
***Primary care***						
Patients with hospitalizations, *n* (%)	19 (47.5%)	296 (54.7%)	.413	75 (57.3%)	309 (60.5%)	.549
Length of stay, median (IQR)	10.5 (19.0)	9.0 (11.0)	.846	10.0 (24.0)	10.0 (18.0)	.516
Patients with readmissions, *n* (%)	5 (11.1%)	82 (15.2%)	.820	15 (11.5%)	91 (17.8%)	.087

a Excluded patients with a first specialist palliative care contact less than 14 days prior death.

b Excluded patients with a first specialist palliative care contact less than 30 days prior death.

**Figure 3:**
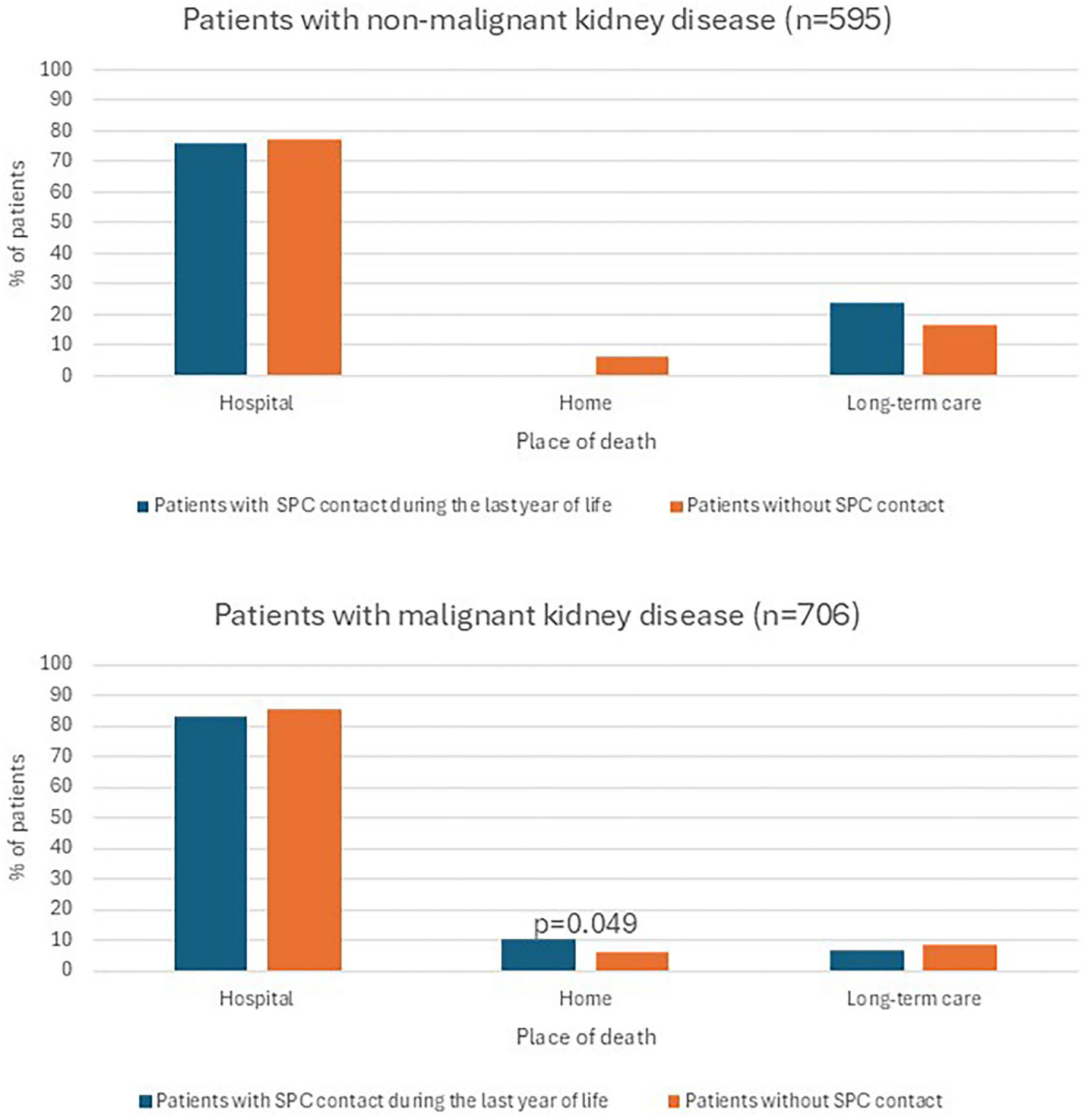
Place of death among patients with contact to specialist palliative care (SPC) services and among those without contact to SPC. Only significant *P*-values are shown.

The location of death was similar among patients who had access to SPC services compared to those without, except for the proportion of deaths at home being higher among patients with malignant kidney disease and SPC service contact (10.6%) when compared to patients without contact (5.9%; *P* = .049). No differences were seen among patients with non-malignant kidney disease (Fig. 3).

## DISCUSSION

Our nationwide retrospective decedent registry study, including all deaths among patients with non-malignant kidney disease and patients with kidney or urothelial cancer in Finland in 2019, revealed that patients with non-malignant kidney disease had low access (<10%) to SPC during the final year of life despite high overall healthcare utilization. This study is among the few to document national disparities in palliative care for this population, exposing persistent inequities compared to patients with malignant conditions [21–24]. In our study, 27.6% of patients with malignant kidney disease received SPC. This was below the global average of access to palliative care among patients with cancer (34%) [22] and below the national average in all patients with cancer in Finland (30%) [17]. However, access to palliative care among patients with malignant kidney disease was comparable to other cohorts of kidney disease patients in the United States (25%) and United Kingdom (27%) [24]. Access for patients with non-malignant kidney disease was even lower compared to those with malignant diseases in our nationwide cohort and substantially lower compared to dialysis cohorts. In a Canadian dialysis cohort, 47% of patients with advanced chronic kidney disease received SPC [26], and in a Medicare cohort of hemodialysis patients, 20% received hospice care at the EOL [27].

Limited access to SPC in non-malignant kidney disease likely reflects multiple factors. Patients with non-malignant kidney diseases are a heterogenous group: older adults with frailty may follow a gradual and slow decline, whereas those on hemodialysis are more likely to have continued decline with intermittent acute worsening of symptoms [28]. Most patients also face a high risk of cardiovascular disease or arrhythmia events at the EOL [29]. This heterogeneity, combined with the lack of standardized referral criteria, makes timely referral to palliative care challenging [30]. Misconceptions that palliative care is synonymous with EOL care, and that its initiation is only appropriate near death, can further deter clinicians from referral [31].

However, a timely referral is of high importance, since in practice, dialysis withdrawal often leaves a narrow window for palliative care, with a median survival of approximately 1 week, or weeks to a few months for patients who have urine output [32, 33].

Internationally, these barriers in making timely referrals, together with the limited prognosis and complex decision-making associated with kidney replacement therapies [34, 35], have led to the development of specialty-aligned palliative care services and integration of palliative care into earlier stages of the illness trajectory, with more seamless transition when EOL care becomes necessary [12, 21].

It is noteworthy that our study demonstrated benefits, in line with other studies (37–40, of SPC services contact among patients with malignant kidney disease, as evidenced by a lower proportion of patients having contacts with the emergency department in secondary care, and a lower proportion being hospitalized in secondary care or readmitted to these services at the EOL. These findings among the patients with malignant disease are most likely due to core elements of palliative care services: advance care planning and planned EOL care pathways directing the care from secondary care to primary care and palliative care wards.

In contrast to patients with malignant disease, the benefits of SPC in reducing secondary care utilization were not evident among patients with non-malignant disease. In this group, patients who received SPC had a longer length of stay in secondary care hospitals and more primary care emergency department than those without SPC contact. This might reflect that patients who were referred to SCP may have had more complex needs or previous repeated hospitalizations that might have prompted the SCP contact. Given that these patients with non-malignant kidney disease were older and had more comorbidities on average, their extended stays may also relate to reduced functional status, poorer prognosis, or more complex medications needed for symptom control.

The third notable finding is the high proportion of patients dying in hospital. On the contrary, patients would often rather die at home or in in hospice [40]. In our study, only eight patients (1.3%) with non-malignant kidney disease died in a SPC ward and none of them died with the support of hospital-at-home service. This proportion is considerably lower than the 10% reported in a cohort from England [41]. Both chronic kidney diseases and cancer are associated with significantly higher hospital costs at the EOL, whereas dying at home is linked with substantial reductions in hospital costs during the final year of life [41]. In our study, among those patients with malignant kidney disease, a higher proportion of patients with SPC service died at home when compared to those without SPC service.

The primary strength of our study lies in its nationwide perspective, relying on data from the Finnish Causes of Death Register and National Care Register providing a validated view beyond individual services and centers [42]. One of its key limitations is the lack of detailed patient characteristics, including demographic and clinical data such as CKD stage or comorbidities. In our study, clinicians reported more contributory causes of death among patients with non-malignant kidney disease: 76% had at least one comorbidity, compared to 60.8% of malignant cases with none. Our cohort was limited to cases where kidney disease was the primary cause of death, despite cardiovascular disease being more common in this population [43]. Events like frailty, falls, and functional decline may compete as primary causes of death [44, 45], suggesting our study may underestimate the broader impact of chronic kidney disease on healthcare utilization. Finally, although no benefit was observed from SPC in health service utilization among patients with non-malignant kidney disease, the limited number of patients receiving such care in our cohort may have dampened potential effects. Future prospective studies should examine both health service utilization and QOL among patients with non-malignant kidney disease.

In conclusion, we demonstrated low access to SPC services among patients with non-malignant kidney disease. Access to SPC guided care toward primary care among patients with malignant kidney disease. Further efforts are needed to educate clinicians about the limited lifespan in patients with non-malignant kidney disease, improving quality of care, and increasing referrals to palliative care.

## Supplementary Material

sfag111_Supplemental_File

## Data Availability

The data that support the findings of this study are available from the Finnish Institute for Health and Welfare, but restrictions apply to the availability of these data, which were used under license for the current study, and so are not publicly available. Data permits can be requested from the Finnish Social and Health Data Permit Authority, Findata (info@findata.fi).
