# Supplementary material for: Access to specialist palliative care and health care service utilization during the final year of life among patients with kidney disease
Source: Clin Kidney J. 2026 Apr 9;19(5):sfag111. doi: 10.1093/ckj/sfag111 (PMC13153784; doi:10.1093/ckj/sfag111)
Supplement: sfag111_Supplemental_File [file sfag111_supplemental_file.docx]

**Supplementary Table 1:** The number of different ICD-10 codes among patients with non-malignant and malignant kidney disease identified from the Causes of Death Register

|  |  | **Number of cases (n)*** |
| --- | --- | --- |
| **Non-malignant kidney diseases** | | |
| E10.2 | Type 1 diabetes mellitus with renal complications | 11 |
| including E10.21 | Type 1 diabetes mellitus with diabetic nephropathy |  |
| including E10.22 | Type 1 diabetes mellitus with diabetic chronic kidney disease |  |
| including E10.29 | Type 1 diabetes mellitus with other diabetic kidney complication |  |
| E11.2 | Type 2 diabetes mellitus with renal complications | 101 |
| including E11.21 | Type 2 diabetes mellitus with diabetic nephropathy |  |
| including E11.22 | Type 2 diabetes mellitus with diabetic chronic kidney disease |  |
| including E11.29 | Type 2 diabetes mellitus with other diabetic kidney complication |  |
| E14.1 | Unspecified diabetes mellitus with  Ketoacidosis | 7 |
| including E14.6 | Unspecified diabetes mellitus with other specified complications |  |
| including E14.8 | Unspecified diabetes mellitus with unspecified complications |  |
| E75.2 | Other sphingolipidosis | 0 |
| I12.0 | Hypertensive chronic kidney disease with stage 5 chronic kidney disease or end stage renal disease (with renal failure) | 122 |
| I12.9 | Hypertensive renal disease without renal failure | 0 |
| I15.0 | Renovascular hypertension | 0 |
| I15.1 | Hypertension secondary to other renal disorders | 0 |
| I13.0 | Hypertensive heart and chronic kidney disease Hypertensive heart and renal disease with (congestive) heart failure | 36 |
| I13.1 | Hypertensive heart and renal disease with renal failure | 27 |
| I13.2 | Hypertensive heart and renal disease with both (congestive) heart failure and renal failure | 215 |
| I13.9 | Hypertensive heart and renal disease, unspecified | 0 |
| N18.1 | Chronic kidney disease (CKD), stage 1 | 0 |
| N18.2 | Chronic kidney disease (CKD), stage 2 | 0 |
| N18.3 | Chronic kidney disease (CKD), stage 3 | 0 |
| N18.4 | Chronic kidney disease (CKD), stage 4 | 0 |
| N18.5 | Chronic kidney disease (CKD), stage 5 | 20 |
| N18.9 | Chronic kidney disease, unspecified | 57 |
| Q61  (including Q61.0-Q61.9) | Cystic kidney disease | 0 |
| **Total non-malignant** |  | 596 |
|  |  |  |
| **Malignant kidney diseases** | | |
| C64 | Malignant neoplasm of kidney, except renal pelvis | 342 |
| C65 | Malignant neoplasm of renal pelvis | 26 |
| C66 | Malignant neoplasm of ureter | 13 |
| C67 | Malignant neoplasm of bladder | 317 |
| C68 | Malignant neoplasm of other and unspecified urinary organs | 9 |
| **Total malignant** |  | **707** |
| All cases |  | **1303** |

**Supplementary Table 2**: ICD-10 codes included in comorbidities

| **Comorbidity** | **Considered if any of the following were listed as contributive cause of death** |
| --- | --- |
| Heart Failure and valvular diseases | I05.0, I05.8, I06.1, I08.0, I08.1, I08.2, I08.3, I08.7, I08.9, I11.0, I11.9, I34.0, I34.1, I34.9, I35.0, I35.1, I35.2, I35.8, I37.0, I36.1, I42.0, I42.1, I42.2, I42.3, I42.4, I42.5, I42.6, I42.7, I42.8, I42.9, I50.0, I50.1, I50.2, I50.3, I50.4, I50.9 |
| Coronary artery disease | I25.0, I25.1, I25.2, I25.5, I25.6, I25.7, I25.8, I25.9 |
| Dementia | F00.0, F00.1, F00.2, F00.9, F01.0, F01.1, F01.2, F01.3, F01.8, F01.0, F01.1, F01.3, F01.8, F01.9, F02.0, F02.1, F02.2, F02.3, F02.4, F02.8, F03, G30.0, G30.1, G30.8, G30.9 |
| Diabetes | E08.0, E08.1, E08.2, E08.3, E08.4, E08.5, E08.6, E08.9, E09.0, E09.1, E09.2, E09.3, E09.4, E09.5, E09.6, E09.9, E10.0, E10.1, E10.2, E10.3, E10.4, E10.5, E10.6, E10.7, E10.9, E11.0, E11.1, E11.2, E11.3, E11.4, E11.5, E11.6, E11.7, E11.8, E11.9, E13.0, E13.1, E13.2, E13.3, E13.3, E13.4, E13.5, E13.6, E13.9,E14.0, E14.1, E14.2, E14.3, E14.4, E14.5, E14.6, E14.7;E14.8, E14.9 |
| Hypertension | I10 |
| Atrial fibrillation or flutter | I48.0, I48.1, I48.2, I48.3, I48.4, I48.9 |
| Stroke or intracerebral hemorrhage | I60.0,I60.1, I60.2, I60.3, I60.4,I60.5,I60.6, I60.7,I60.8,I60.9 I61.0, I61.1,I61.2, I61.3,I61.4, I61.5,I61.6, I61.8, I61.9, I63.0, I63.1, I63.2, I63.4, I63.5, I63.6, I63.8, I63.9, I67.6, I67.9, I69.0, I69.1, I69.4 |
| Peripherial artery disease | I70.2, I73.9 |
| Chronic obstructive lung disease | J43.9, J44.0, J44.1, J44.8, J44.9 |
| Cancer | C00-C96 |
| Chronic kidney disease | N18.1, N18.2, N18.3, N18.4, N18.5,N18.6, N18.9 |
| Infections diseases | A00-B99, J06.9, J15.4, J15.9, J18.0, J18.9, J22, J86.9, N39.0, K37, K65.8, K81.0, K85.4, L023, L024, M86.9, N10 |

**Supplementary Table 3:** Patient characteristics among those who had contact to SPC services compared to those without

|  | **Non-malignant kidney disease** | |  | **Malignant kidney disease** | |  |
| --- | --- | --- | --- | --- | --- | --- |
|  | **Patients with Contact to Specialist Palliative Care** | **Patients with No Contact to Specialist Palliative Care** |  | **Patients with Contact to Specialist Palliative Care** | **Patients with No Contact to Specialist Palliative Care** |  |
| n | 54 | 541 |  | 195 | 511 |  |
| Females, n (%) | 37 (68.5%) | 289 (53.4%) | 0.044 | 69 (35.4%) | 180 (35.2%) | 1.000 |
| Age at death (mean±SD) | 83.0±12 | 86.0±12 | 0.533 | 75.0±17 | 79.0±15 | 0.120 |
|  |  |  |  |  |  |  |
| **Municipality type at time of death** |  |  |  |  |  |  |
| Urban | 45 (83.3%) | 307 (56.7%) | <0.001 | 162 (83.1%) | 297 (58.1%) | <0.001 |
| Semi-urban | 3 (5.6%) | 107 (19.8%) | 0.009 | 16 (8.2%) | 116 (22.7%) | <0.001 |
| Rural | 6 (11.1%) | 127 (23.5%) | 0.039 | 17 (8.7%) | 98 (19.2%) | <0.001 |
|  |  |  |  |  |  |  |
| Had home care during the final year of life | 45 (83.3%) | 413 (76.3% | 0.309 | 141 (72.3%) | 310 (60.7%) | 0.004 |
|  |  |  |  |  |  |  |
| **Prevalence of contributive causes of death*** |  |  |  |  |  |  |
| None | 19 (35.2%) | 126 (23.3%) | 0.066 | 137 (70.3%) | 292 (57.1%) | 0.001 |
| Dementia | 6 (11.1%) | 89 (16.5%) | 0.435 | 12 (6.2%) | 39 (7.6%) | 0.626 |
| Infectious diseases | 3 (5.6%) | 64 (12.0%) | 0.183 | 3 (1.5%) | 19 (3.7%) | 0.223 |
| Diabetes | 8 (14.8%) | 72 (13.3%) | 0.681 | 8 (4.1%) | 27 (5.3%) | 0.698 |
| Hypertension | 3 (5.6%) | 52 (9.6%) | 0.461 | 9 (4.6%) | 34 (6.7%) | 0.380 |
| Coronary artery disease | 5 (9.3%) | 24 (4.4%) | 0.171 | 14 (7.2%) | 49 (9.6%) | 0.377 |
| Heart Failure and valvular diseases | 2 (3.7%) | 71 (13.1%) | 0.048 | 2 (1.0%) | 24 (4.7%) | 0.023 |
| Atrial fibrillation or flutter | 4 (7.4%) | 84 (15.5%) | 0.157 | 7 (3.6%) | 19 (3.7%) | 1.000 |
| Stroke or Intracerebral hemorrhage | 4 (7.4%) | 34 (6.3%) | 0.768 | 5 (2.6%) | 16 (3.1%) | 0.808 |
| Peripherial artery disease | 2 (3.7%) | 21 (3.9%) | 1.000 | 1 (0.5%) | 7 (1.4%) | 0.456 |
| Chronic Obstructive Pulmonary Disease | 2 (3.7%) | 23 (4.3%) | 1.000 | 7 (3.6%) | 19 (3.7%) | 1.000 |
| Cancer | 3 (5.6%) | 35 (6.5%) | 1.000 | 8 (4.1%) | 35 (6.8%) | 0.218 |
| Chronic kidney disease | 0 | 9 (1.7%) | 1.000 | 5 (2.6%) | 12 (2.3%) | 0.791 |
| Palliative care identification (defined as prevalence of ICD-10 code Z51.5) by the time of death | 12 (22.2%) | 45 (8.3%) | 0.003 | 163 (83.6%) | 222 (43.4%) | <0.001 |
|  |  |  |  |  |  |  |
| **Place of death** |  |  |  |  |  |  |
| Hospital* | 41 (75.9%) | 417 (77.1%) | 0.866 | 162 (83.1%) | 436 (85.3%) | 0.483 |
| At the palliative care ward at the time of death (included in hospital*) | 8 (14.8%) | 0 | <0.001 | 42 (21.5%) | 0 | <0.001 |
| Home | 0 (0%) | 33 (6.1%) | 0.062 | 20 (10.3%) | 30 (5.9%) | 0.049 |
| Died with specialist palliative hospital at home care | 0 | 0 | n.a | 8 (4.1%) | 0 | <0.001 |
| Long term care facility | 13 (24.1%) | 91 (16.8%) | 0.189 | 13 (6.7%) | 45 (8.8%) | 0.444 |
